# Supplementary material for: Synthesis and Evaluation of Two Long-Acting SSTR2 Antagonists for Radionuclide Therapy of Neuroendocrine Tumors
Source: Pharmaceuticals (Basel). 2022 Sep 16;15(9):1155. doi: 10.3390/ph15091155 (PMC9503898; doi:10.3390/ph15091155)
Supplement: Supplementary file 1 [file pharmaceuticals-15-01155-s001.zip › pharmaceuticals-1889610-supplementary.pdf]

Synthesis and Evaluation of Two Long-Acting SSTR2 Antagonists for Radionuclide Therapy of Neuroendocrine Tumors

Sofia Koustoulidou [1,†], Maryana Handula [1,†], Corrina de Ridder [1], Debra Stuurman [1], Savanne Beekman [1], Marion de Jong [1,‡], Julie Nonnekens [1,2] and Yann Seimbille [1,3,*]

[1] Department of Radiology and Nuclear Medicine, Erasmus MC Cancer Institute, Erasmus University Medical Center Rotterdam, 3015 GD Rotterdam, The Netherlands
[2] Department of Molecular Genetics, Erasmus MC Cancer Institute, Erasmus University Medical Center Rotterdam, 3015 GD Rotterdam, The Netherlands
[3] Life Sciences Division, TRIUMF, Vancouver, BC V6T 2A3, Canada
* Correspondence: y.seimbille@erasmusmc.nl; Tel.: +31-10-703-8961
† These authors contributed equally to this work.
‡ The author recently passed away.

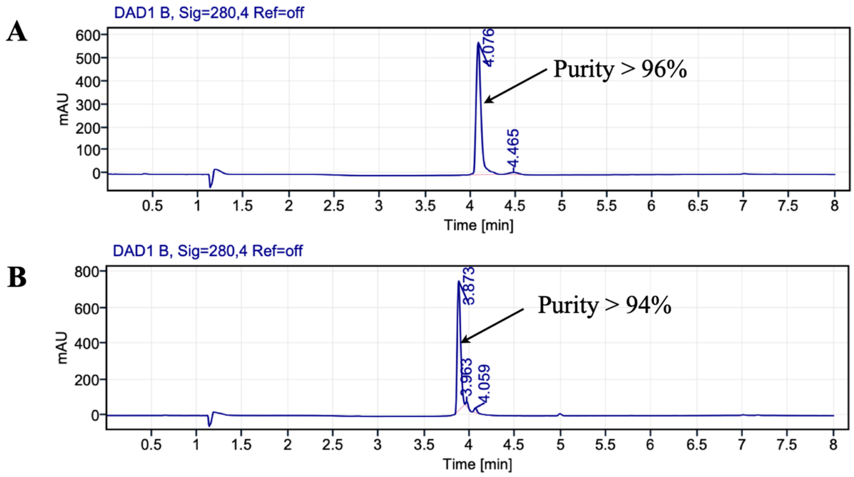

Figure S1. HPLC chromatograms of A) compound **8a** and B) compound **8b**.

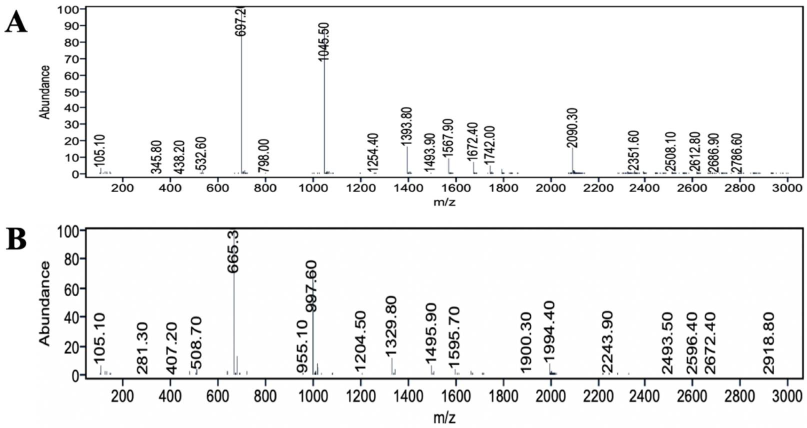

Figure S2. Mass spectra of A) compound **8a** and B) compound **8b**.

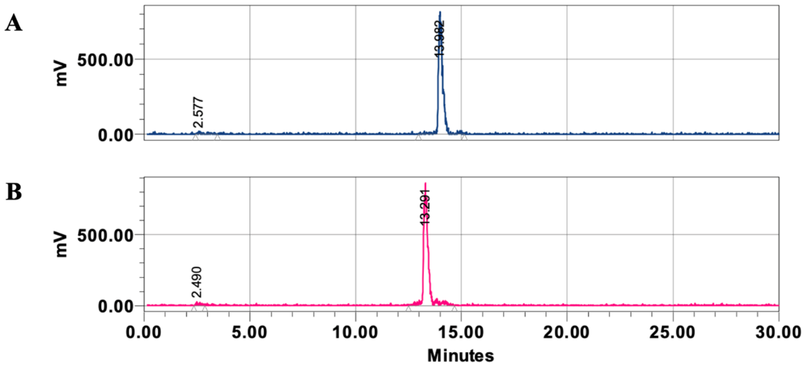

Figure S3. Radio-HPLC chromatograms of A) [^{177}Lu]Lu-**8a** and B) [^{177}Lu]Lu-**8b**.

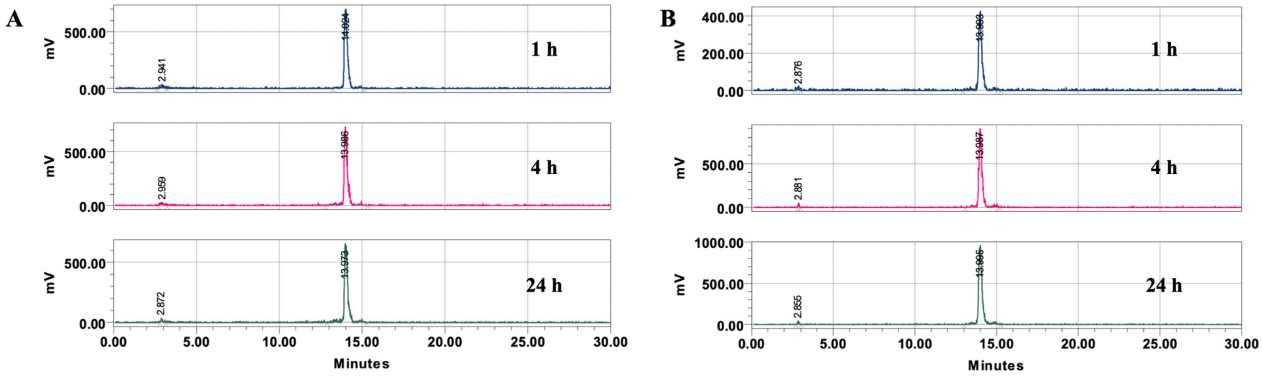

Figure S4. Radio-HPLC chromatograms of the stability studies performed for [^{177}Lu]Lu-**8a** at 1 h, 4 h and 24 h post incubation at 37 °C of the radioligand in A) PBS and B) mouse serum.

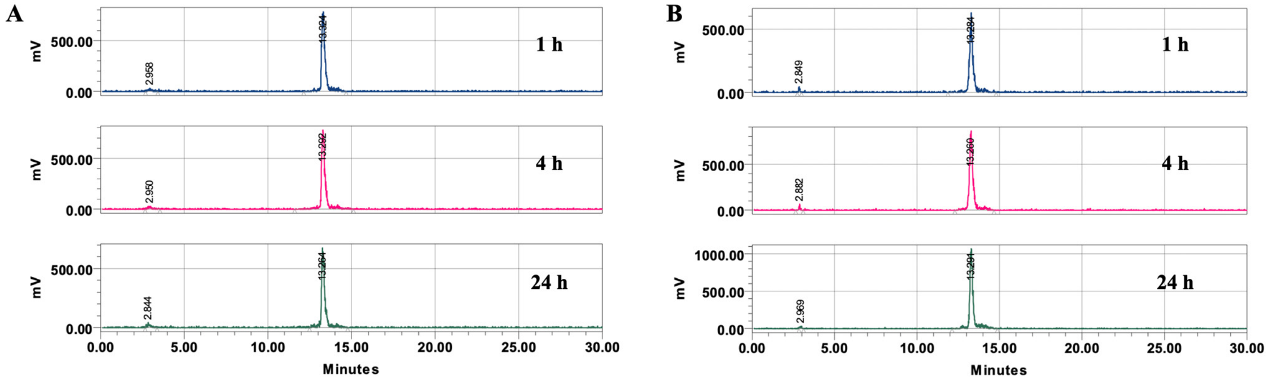

Figure S5. Radio-HPLC chromatograms of the stability studies performed for [^{177}Lu]Lu-**8b** at 1 h, 4 h and 24 h post incubation at 37 °C of the radioligand in A) PBS and B) mouse serum.

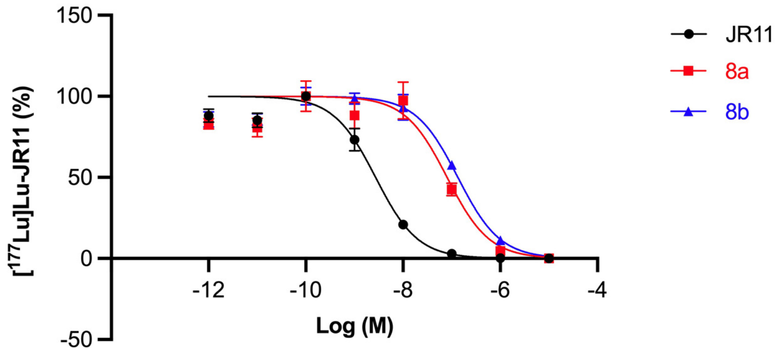

Figure S6. IC_{50} curves of the *in vitro* competition binding assay for compounds **8a** and **8b** in increasing concentrations (M) while [^{177}Lu]Lu-JR11 was used as radioligand.

Table S1. *Ex vivo* biodistribution analysis of [^{177}Lu]Lu-**8a** (20 MBq/0.5 nmol) at 4, 24, 48 and 72 h post-injection (n = 4 mice/group). Data is represented as percentage of injected dose per gram of tissue (% ID/g).

Organ	4 h	24 h	24 h - Block	48 h	72 h
Blood	21.3 ± 1.1	18.1 ± 4.2	14.9 ± 2.1	12.9 ± 1.2	8.6 ± 0.5
Tumor	4.1 ± 0.5	6.5 ± 0.2	5.2 ± 0.6	7.3 ± 0.4	7.3 ± 0.9
Heart	7.6 ± 1.2	5.1 ± 0.7	5.1 ± 0.6	4.8 ± 0.5	3.9 ± 0.7
Lung	9.9 ± 2.9	7.5 ± 0.6	5.4 ± 0.6	7.2 ± 0.3	6.0 ± 0.5
Liver	4.6 ± 1.2	3.7 ± 0.2	2.9 ± 0.5	3.6 ± 0.2	2.9 ± 0.3
Spleen	3.2 ± 0.7	3.8 ± 0.4	3.6 ± 0.6	5.8 ± 0.6	5.4 ± 0.2
Stomach	3.8 ± 0.4	3.1 ± 1.4	1.7 ± 0.4	3.3 ± 1.2	3.0 ± 0.5
Intestine	2.5 ± 0.2	1.7 ± 0.1	1.3 ± 0.2	1.4 ± 0.1	1.1 ± 0.0
Pancreas	7.2 ± 0.3	6.6 ± 0.3	2.4 ± 0.4	6.3 ± 0.7	5.3 ± 1.2
Kidneys	9.6 ± 1.2	18.4 ± 1.9	19.7 ± 1.4	22.6 ± 1.9	21.6 ± 1.4
Muscle	0.8 ± 1.1	1.1 ± 0.1	0.8 ± 0.1	1.2 ± 0.5	0.8 ± 0.1
Skin	5.9 ± 1.1	5.4 ± 0.9	3.2 ± 1.2	6.3 ± 1.6	4.8 ± 0.3
Bone	1.3 ± 1.8	1.5 ± 1.1	1.5 ± 0.3	0.9 ± 0.4	1.2 ± 1.0
Bone Marrow	1.2 ± 2.0	-0.2 ± 0.1	-0.8 ± 1.7	-0.3 ± 0.1	-0.1 ± 0.1

Table S2. *Ex vivo* biodistribution analysis of [^{177}Lu]Lu-JR11 (20 MBq/0.5 nmol) at 4, 24, 48 and 72 h post-injection (n = 4 mice/group). Data is represented as percentage of injected dose per gram of tissue (% ID/g).

Organ	4 h	24 h	24 h - Block	48 h	72 h
Blood	-0.1 ± 0.0	-0.1 ± 0.1	-0.1 ± 0.1	0.0 ± 0.0	-0.1 ± 0.0
Tumor	8.4 ± 0.5	6.1 ± 0.5	0.4 ± 0.0	4.6 ± 0.3	3.6 ± 0.4
Heart	0.0 ± 0.0	0.0 ± 0.0	0.0 ± 0.0	0.0 ± 0.0	-0.1 ± 0.0
Lung	1.3 ± 0.3	0.4 ± 0.0	0.1 ± 0.0	0.2 ± 0.0	0.1 ± 0.0
Liver	0.5 ± 0.0	0.3 ± 0.0	0.3 ± 0.0	0.2 ± 0.0	0.2 ± 0.0
Spleen	0.3 ± 0.1	0.1 ± 0.0	0.0 ± 0.0	0.0 ± 0.0	0.0 ± 0.0
Stomach	1.7 ± 0.3	1.5 ± 1.2	0.1 ± 0.1	0.6 ± 0.2	0.4 ± 0.1
Intestine	0.5 ± 0.1	0.2 ± 0.0	0.1 ± 0.0	0.1 ± 0.0	0.1 ± 0.0
Pancreas	3.5 ± 0.4	0.9 ± 0.3	0.1 ± 0.0	0.6 ± 0.1	0.3 ± 0.0
Kidneys	12.6 ± 1.2	8.6 ± 2.3	5.9 ± 1.2	4.1 ± 0.2	3.4 ± 1.4
Muscle	-0.1 ± 0.0	-0.1 ± 0.0	-0.1 ± 0.0	-0.1 ± 0.0	-0.1 ± 0.0
Skin	0.3 ± 0.1	0.0 ± 0.1	-0.1 ± 0.1	-0.2 ± 0.1	-0.4 ± 0.2
Bone	0.1 ± 0.0	-0.6 ± 0.4	-0.4 ± 0.5	-0.2 ± 0.2	-0.1 ± 0.1
Bone Marrow	1.1 ± 2.0	0.1 ± 0.0	0.2 ± 0.0	0.1 ± 0.0	0.1 ± 0.0
